# Queries Suggested by the Perusal of Mr. Kington's Case of Luxation of the Jaw

**Published:** 1814-05

**Authors:** 


*For the Medical and Physical Journal.*

### *Queries suggested by the Perusal of Mr. Kington's Case of Luxation of the Jaw.*

HAVING perused the case of Luxation of the Jaw, published by Mr. A. Kington, in your valuable Journal, I would candidly ask the following query:—What was Mr. Kington's motive for bleeding per ulna, or by leeches upon the part affected, or both, immediately after he found the swelling and inflammation so great, and the muscles so strongly in action, as to render it impracticable to reduce it without doing great injury? and why he, the next day, when he found his patient's symptoms so much increased, did not bleed to a larger extent, as he states his patient to have been of a strong muscular habit? Might not the jaw have been much sooner reduced, and the patient put out of his misery, if from twenty to thirty ounces of blood had been directly drawn, and the warm bath used, &c.?

